# Supplementary material for: MetaRibo-Seq measures translation in microbiomes
Source: Nat Commun. 2020 Jun 29;11:3268. doi: 10.1038/s41467-020-17081-z (PMC7324362; doi:10.1038/s41467-020-17081-z)
Supplement: Supplementary file 10 — Supplementary Data 7 [file 41467_2020_17081_MOESM10_ESM.zip › File2/Confidence_High_Taxonomy/86445_out.krona.html]

Javascript must be enabled to view this page.

members
magnitude
magnitudeUnassigned
count
unassigned
taxon
rank

86445\_out

3

3
superkingdom
2

3
201174
phylum

3
1760
class

order
85004
3

31953
family
3

3
genus
1678

species

SRS014979\_contig\_number\_contig-100\_27264.121395
78343
1

2

SRS1041031\_contig\_number\_7977SRS150029\_contig\_number\_12360
1680
species
